# Genome-Wide Detection of Copy Number Variants in Chinese Indigenous Horse Breeds and Verification of CNV-Overlapped Genes Related to Heat Adaptation of the Jinjiang Horse

**DOI:** 10.3390/genes13040603

**Published:** 2022-03-28

**Authors:** Min Wang, Yu Liu, Xiaokun Bi, Hongying Ma, Guorong Zeng, Jintu Guo, Minghao Guo, Yao Ling, Chunjiang Zhao

**Affiliations:** 1 College of Animal Science and Technology, China Agricultural University, Beijing 100193, China; min@cau.edu.cn (M.W.); yliu316@cau.edu.cn (Y.L.); xiaokunbi@cau.edu.cn (X.B.); lingzi@cau.edu.cn (Y.L.); 2 Equine Center, China Agricultural University, Beijing 100193, China; 3 Key Laboratory of Animal Genetics, Breeding and Reproduction, Ministry of Agriculture, Beijing 100193, China; 4 National Engineering Laboratory for Animal Breeding, Beijing 100193, China; 5 Beijing Key Laboratory for Genetic Improvement of Livestock and Poultry, Beijing 100193, China; 6 Shaanxi Key Laboratory for Animal Conservation, Shaanxi Institute of Zoology, Xi’an 710032, China; mhying7916@163.com; 7 Jinjiang Animal Husbandry and Veterinary Station, Quanzhou 362200, China; 13655922266@163.com (G.Z.); minwang0107@163.com (J.G.); guominghao_1325@163.com (M.G.)

**Keywords:** copy number variants, Chinese indigenous horse breeds, Jinjiang horse, heat adaptation

## Abstract

In the present study, genome-wide CNVs were detected in a total of 301 samples from 10 Chinese indigenous horse breeds using the Illumina Equine SNP70 Bead Array, and the candidate genes related to adaptability to high temperature and humidity in Jinjiang horses were identified and validated. We determined a total of 577 CNVs ranging in size from 1.06 Kb to 2023.07 Kb on the 31 pairs of autosomes. By aggregating the overlapping CNVs for each breed, a total of 495 CNVRs were detected in the 10 Chinese horse breeds. As many as 211 breed-specific CNVRs were determined, of which 64 were found in the Jinjiang horse population. By removing repetitive CNV regions between breeds, a total of 239 CNVRs were identified in the Chinese indigenous horse breeds including 102 losses, 133 gains and 4 of both events (losses and gains in the same region), in which 131 CNVRs were novel and only detected in the present study compared with previous studies. The total detected CNVR length was 41.74 Mb, accounting for 1.83% of the total length of equine autosomal chromosomes. The coverage of CNVRs on each chromosome varied from 0.47% to 15.68%, with the highest coverage on ECA 12, but the highest number of CNVRs was detected on ECA1 and ECA24. A total of 229 genes overlapping with CNVRs were detected in the Jinjiang horse population, which is an indigenous horse breed unique to the southeastern coast of China exhibiting adaptability to high temperature and humidity. The functional annotation of these genes showed significant relation to cellular heat acclimation and immunity. The expression levels of the candidate genes were validated by heat shock treatment of various durations on fibroblasts of horses. The results show that the expression levels of *HSPA1A* were significantly increased among the different heat shock durations. The expression level of *NFKBIA* and *SOCS4* declined from the beginning of heat shock to 2 h after heat shock and then showed a gradual increase until it reached the highest value at 6 h and 10 h of heat shock, respectively. Breed-specific CNVRs of Chinese indigenous horse breeds were revealed in the present study, and the results facilitate mapping CNVs on the whole genome and also provide valuable insights into the molecular mechanisms of adaptation to high temperature and humidity in the Jinjiang horse.

## 1. Introduction

Copy number variations (CNVs) are a type of genome structure variation in which genomic segments range from 50 bp to several Mb, and they are affected by large-scale insertions, deletions, duplications, inversions and translocations relative to the reference genome [1]. CNVs might be one of the main factors affecting phenotypic diversity and evolutionary adaptation in animals, employing a wide variety of mechanisms, such as gene dosage and transcript structure alterations, to modulate organismal plasticity [2]. Previous studies on other mammals have hypothesized that CNVs with high-frequency differences among breeds are involved in population-specific selection and adaptation to the environment [3,4,5]. In horses, many studies have reported the discovery of CNVs in the whole genome, which were associated with equine diseases [6], adaptations and phenotypic traits. Wang et al. [7] found seven genes in candidate CNV regions, which may have some relation to the adaption to the severe environment of plateaus in Chinese indigenous horses. Previous studies have shown that body size in horses is mainly associated with homozygous or heterozygous CNVs on ECA1, ECA8 and ECA9 [8]. A 4.6 kb duplication in intron 6 of the STX17 gene on ECA25 has been proposed to be the causative mutation of the gray phenotype, a progressive hair depigmentation syndrome accompanied by an increased susceptibility to melanoma [9].

The combination of high temperature and humidity is a serious challenge for all animals, which may induce physiological changes and lead to low reproductive and production performance during the hot seasons. Especially for horses, high temperature and humidity seriously affect their sports performance. The manifestations of heat exhaustion syndromes in horses performing submaximal exercise over distances under high temperature and humidity have been well documented [10,11,12]. In addition, exertional heat illness (EHI) is more likely to affect horses in very hot and humid weather conditions [12,13]. A progressive increase in the prevalence of horse EHI casualties is anticipated due to global warming in the future [14,15]. Thus, the identification of specific genes conferring thermotolerance in heat-tolerant breeds may be an additional strategy to improve the genetic background in sport breeds. The Jinjiang horse is an excellent indigenous breed in the coastal areas of southwest China. Under the influence of the environment and animal husbandry practices, the Jinjiang horse has developed a strong adaptation to high-temperature and humidity environments. A comprehensive study on the genetics of the Jinjiang horse offers an opportunity to not only understand the molecular basis of adaptation to high temperature and high humidity, but also accelerate breeding efforts of the Jinjiang horse.

The objective of this study was to construct a genome-wide CNV map of Chinese indigenous horses using genotyping data of genomic SNPs. In addition, we focused on the analysis of the Jinjiang horse population to reveal the candidate genes related to high temperature and humidity adaptation. Candidate genes were validated at the cellular level to gain a more comprehensive understanding of the molecular mechanisms in the adaptation to high temperature and humidity. Our study provides novel information on the genome-wide CNVs of Chinese indigenous horses and also facilitates the conservation and breeding activities of the Jinjiang horse.

## 2. Materials and Methods

### 2.1. Preprocessing the Source Data

The dataset of horse populations in this study was derived from our previously published data, with the accession code GSE128376. The data comprise a total of 301 animals from 10 Chinese indigenous breeds genotyped with the Illumina Equine SNP70 Bead Array from a study of Ma et al. [16]. The sampled horse breeds included the Mongolian horse (IMG) from Inner Mongolia (representing the Mongolia breed group); the Daan horse (DA) from Jilin Province (belonging to the Mongolia horse group); the Chakouyi horse (CKY) from Gansu Province (representing the Hequ horse group); the Naqu horse (NQ) from Tibet (standing for the Tibet horse group); the Kazakh horse (KZK) from Xinjiang (representing the Kazakh horse group), and horse breeds from South China mainly belonging to the Southwest horse group, which consisted of the Baise horse (BS) from Guangxi Province, the Tengchong horse (TC), the Zhaotong horse (ZT), the Lijiang horse (LJ) from Yunnan Province and the Jinjiang horse (JJ) from Fujian Province, which was the only indigenous breed from Southeast China. Detailed information is shown in Table 1.

### 2.2. Genome-Wide Detection of CNVs and CNVRs

We extracted all genotype calls from the raw intensity data for each sample using GenomeStudio software version 2.0 (Illumina Inc., San Diego, CA, USA). Quality control of the raw data was carried out with following conditions: (1) individuals with a call rate less than 90% were excluded; (2) the loci with a call frequency less than 90% of SNPs were excluded. Subsequently, the signal intensity data of the LRR (log R ratio) and BAF (B allele frequency) for each SNP were exported from Illumina GenomeStudio software. A total of 282 individuals passed the quality control for the subsequent CNV calling (Table 1).

The penncnv algorithm was applied to detect individual-based CNVs with a hidden Markov model [17]. The sex chromosomes were excluded from the analysis because the penncnv algorithm was only applied to autosomes. After obtaining the initial CNVs, quality control was performed and individuals meeting the following conditions were retained: LRR standard deviation less than 0.35; BAF drift less than 0.01; and WF (waviness factor) caused by GC contents less than 0.05. In order to ensure the reliability of CNV detection, only CNVs with a length greater than 1Kb and containing at least 5 SNPs were retained.

Based on the CNVs detected with the penncnv algorithm, CNVRuler software was used to determined copy number variation regions (CNVRs) using a reciprocal overlap strategy. There was at least one SNP overlap between CNVs until the overlap of the merged CNV and other CNVs was less than 50%. For the detection of breed-specific CNVRs, we defined any CNVR identical between two or more horses across breeds as a shared CNVR, while any CNVRs that were detected in only a single breed and not shared with any other breeds were defined as breed-specific CNVRs. Thereafter, we summarized the genome-wide distribution of CNVRs in Chinese indigenous breeds by removing the overlapping CNVRs among breeds.

We compared our results with the reported results of ten existing horse CNV studies [6,7,8,18,19,20,21,22,23,24], to verify the reliability of our study. Because the data were analyzed based on the EquCab2.0 reference genome assembly in several studies, we converted their results to coordinates on the EquCab3.0 reference genome assembly using the NCBI Genome Remapping Service (https://www.ncbi.nlm.nih.gov/genome/tools/remap, accessed on 8 June 2021). Subsequently, the comparisons of the CNVRs on autosomes were computed using the genomic intersection functionality from BEDTools [25].

### 2.3. Gene Annotation and Enrichment Analysis

We used BioMart (http://www.biomart.org/, accessed on 10 June 2021) in the Ensemble database to identify the genes located within or partially overlapping with the CNVRs in the Jinjiang horse [26]. To further investigate the function of these CNVRs, functional enrichment analysis of the CNVR-harboring genes was performed using human orthologs. Analyses with the Kyoto Encyclopedia of Genes and Genomes (KEGG) pathway and Gene Ontology (GO) of protein-coding genes were carried out using the KOBAS software (version 3.0) [27].

### 2.4. Validation of Genes Overlapping with the CNVRs in Jinjiang Horses

#### 2.4.1. Cell Culture and Heat Shock Treatment

Horse fibroblasts (generous gift from Prof. Xingbo Zhao of the China Agricultural University) were cultured in Dulbecco’s modified Eagle’s medium (DMEM) containing penicillin (100 IU/mL) and 10% fetal bovine serum (Invitrogen, Carlsbad, CA, USA). The cells were cultured at 37 °C in a humidified atmosphere containing 5% CO_2_. Cells were placed in fresh medium 24 h before heat shock. At approximately 80% confluence, the cells were transferred to an incubator with 5% CO_2_ at 42 °C for a further 10 h, and the medium and cells were collected at 2 h intervals during the incubation.

#### 2.4.2. RNA Isolation and Reverse Transcription

Cells collected at different durations of the heat shock were snap-frozen in liquid nitrogen, and total RNA was isolated using TRIzol reagent (#15596026, Thermo Fisher Scientific, Waltham, MA, USA). Removal of potential genomic DNA contamination and reverse transcription of total RNA to cDNA were achieved using a commercially available kit according to the manufacturer’s protocol (FastKing RT Kit With gDNase, Tiangen Biotech, Beijing, CHN). All cDNA samples were stored at −80 °C.

#### 2.4.3. Real-Time Quantitative PCR

Genes related to heat shock were selected for gene expression analyses based on the CNVR results and functional annotation of the Jinjiang horse. Candidate genes were chosen based on their biological relevance to cellular heat acclimation, regulation of the cellular response to heat or evidence of the genes related to heat shock and adaptation to high-temperature and humidity environments in previous studies. The primers for qPCR were designed using Primer-BLAST, and the reference sequences were retrieved from GenBank (both available online at https://www.ncbi.nlm.nih.gov, accessed on 16 August 2021). The primer sequences are listed in Table S1.

Relative quantification of the transcript levels was performed using a SuperReal PreMix Plus (SYBR Green) (#FP205, Tiangen Biotech, Beijing, CHN). The reactions were performed at 95 °C for 15 min and then for 30 cycles at 95 °C for 15 s, 60 °C for 5 s and 72 °C for 2 min. The melting curve was analyzed to determine the specific amplification of the products, and all reactions were performed in three repetitions. The effects of six different heat shock duration levels ranging from 0 h to 10 h on gene expression in cells were evaluated with the housekeeping gene *ACTB* as an endogenous reference. The cycle threshold (Ct) values of the target genes were normalized to the median Ct value of the *ACTB* reference gene, and then the fold changes were calculated using the 2^−ΔΔ*C*^_T_ equation [28]. Data were statistically analyzed by one-way ANOVA. qRT-PCR products were sequenced to assure their specificity, and all presented equine-specific similarity (BLAST analysis).

## 3. Results

### 3.1. Genome-Wide Detection of CNVs in Ten Chinese Indigenous Horse Breeds

After filtering, a total of 63,587 SNPs and 282 individuals remained for the subsequent CNV calling. After applying a stringent CNV calling pipeline, we determined a total of 577 CNVs on 31 pairs of autosomal chromosomes (Table 2 and Table S2). On average, each individual had 2.05 CNV events. The length of these CNVs varied in different breeds, ranging from 1.06 Kb to 2023.07 Kb, and the average length of CNVs per breed was 261.73 Kb. We found the largest number of CNVs (134) in the Jinjiang horse population, including 72 breed-specific CNVs. The average number of individual CNVs identified in the Zhaotong horse population was the largest (2.68), while the Jinjiang horse population had the largest average number of individual breed-specific CNVs (1.31).

### 3.2. Diversity of CNVRs in Breed Clusters

**Figure 1:**
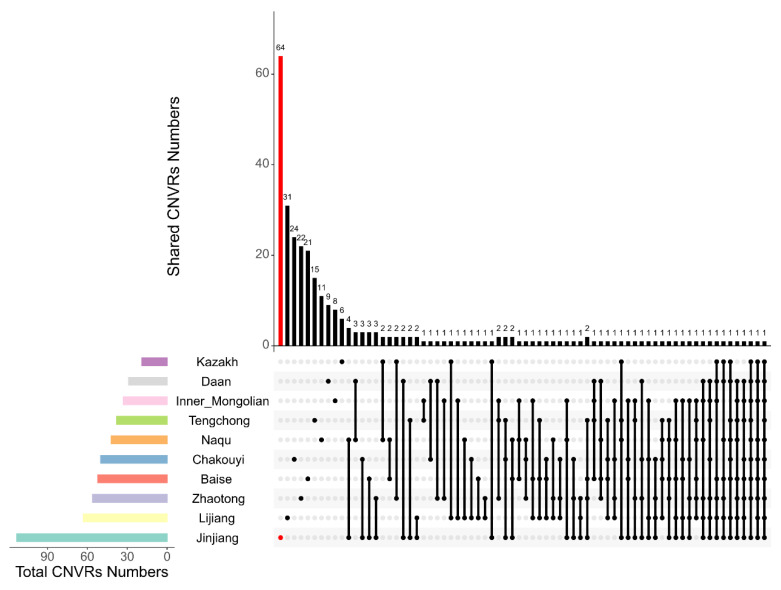
Breed-specific CNVR analysis across the ten horse breeds. The lower left bar chart shows the total amount of CNVRs contained in each original CNVR dataset (classified by horse breeds). In the lower right chart, the first 10 dots indicate the corresponding horse breeds on the left, and their breed-specific CNVR numbers are shown in the upper bar chart. The shared CNVRs between or among breeds are indicated with the vertical lines connecting the dots which represent the breeds on the left, and the numbers of CNVRs shared by the connected breeds are shown in the upper bar chart.

After merging overlapping CNVs, a total of 495 CNVRs were detected on 31 pairs of autosomal chromosomes in the 10 Chinese horse breeds. On average, each horse breed had 50 CNVRs. The Jinjiang horse population had the highest number of CNVRs (113), and the Kazak horse population had the lowest number of CNVRs (19). Detailed information is shown in Table 3 and Table S2. We defined any CNVRs that were detected in only a single breed and not shared with any other breeds as breed-specific CNVRs. Among the 495 CNVRs, 211 (42.6%) were breed-specific CNVRs, of which 64 were found in the Jinjiang horse population, followed by Lijiang horses with 31 breed-specific CNVRs (Figure 1). In addition, among the CNVRs found in these 10 breeds, 44 CNVRs were shared among 3 or more breeds.

**Figure 2:**
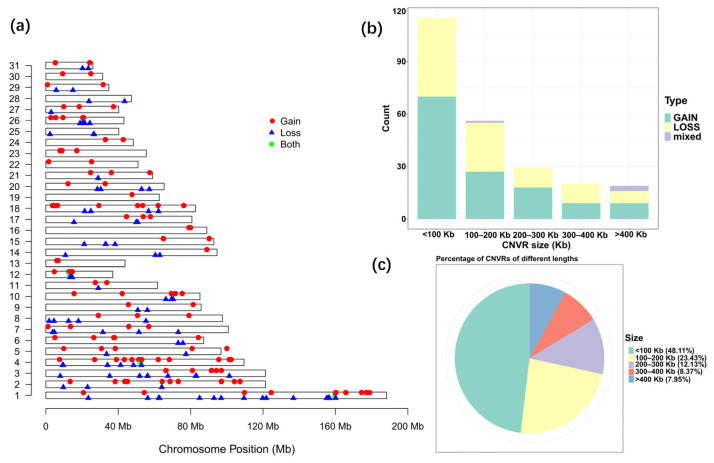
Statistics of CNVRs on equine autosomes. (**a**) Map of CNVRs in the horse genome. Red, blue and green represent gain, loss and both (gain and loss), respectively. (**b**) Size range distribution of the CNVRs detected. (**c**) Scale distribution of the CNVRs detected.

Due to the existence of shared CNVRs between different breeds, a more comprehensive CNVR map and a statistical summary for the indigenous horse genomes were constructed by removing repetitive CNV regions among breeds. A total of 239 CNVRs were generated for the indigenous horse genomes after removing duplicate CNVRs, including 102 losses, 133 gains and 4 of both events (losses and gains in the same region) (Table S2). The statistics of all CNVRs are shown in Table 4. The detected CNVR length varied greatly from 1.06 Kb to 2438.55 Kb, with an average length of 174.65 Kb. The total detected CNVR length was 41.74 Mb, accounting for 1.83% of the total length of horse autosomal chromosomes. There were 115 CNVRs smaller than 100 Kb, accounting for 48.11% of the total number of CNVRs, and 23.43% (56) of CNVRs ranged from 100 to 200 Kb, while only 7.95% of CNVRs were larger than 400 Kb (Figure 2b,c).

The distribution frequency of CNVRs varied on different chromosomes. In general, the longer the chromosome, the higher the frequency and density of the CNVR distribution. The coverage of CNVRs on each chromosome varied from 0.47% to 15.68%, with the highest coverage on ECA 12 (Table 4, Figure 2a). Remarkably, CNVRs were not evenly distributed on all chromosomes. The largest number of CNVRs was detected on ECA 1, followed by 24 detected on ECA 4, and only 1 found on ECA 19.

### 3.3. Comparison with Other Studies on CNVRs in Horse

We compared the predicted CNVRs in this study with those reported in ten previous studies using various platforms (Table 5) [6,7,8,18,19,20,21,22,23,24]. A total of 108 (45.19%) CNVRs were the same as those previously reported, and the remaining 131 (54.81%) CNVRs were novel (Table S2). This suggests that nearly half of the CNVRs that we detected here were verified by other studies.

### 3.4. Functional Annotation of the CNVRs in Jinjiang Horses

Considering the unique adaptability of the Jinjiang horse population to high temperature and humidity, we annotated the CNVRs identified in the Jinjiang horse population. Based on the Ensembl [26] annotation of the EquCab 3.0 genome, 229 genes overlapped with all 113 CNVRs of the Jinjiang horse including breed-specific CNVRs and CNVRs shared with other breeds, as shown in Table S3. These genes included 215 protein-coding genes, as well as some lncRNA genes, miRNA genes, small nucleolar genes (snoRNA) and processed pseudogenes (Table S3).

**Figure 3:**
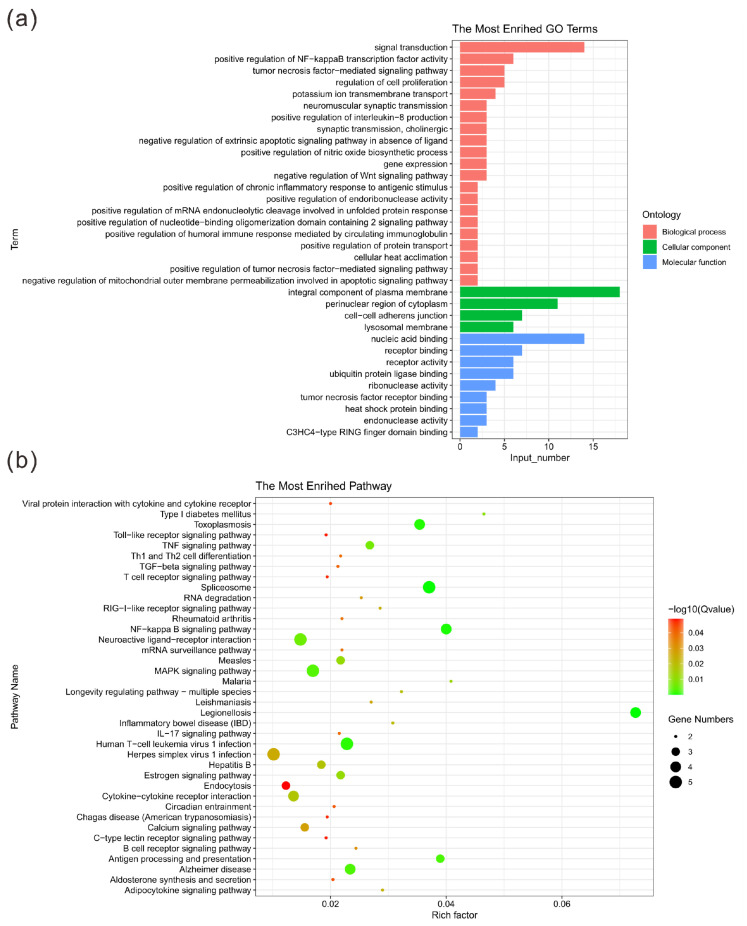
Statistics of GO terms and pathways. (**a**) Histogram of the top 30 GO terms. The ordinate shows the enriched GO term; the abscissa shows the number of genes in the term. Orange, green and blue indicate biological processes, cellular components and molecular functions, respectively. (**b**) Bubble diagram of the top 20 pathways. The pathway names are shown in the legend on the left. The abscissa is the enrichment factor, which represents the ratio of the proportion of genes annotated to a pathway in a differential gene to the proportion of genes in all genes annotated to that pathway. The larger the enrichment factor, the more significant the level of enrichment of the differentially expressed genes in this pathway. The size and color of the dots represent the number of enriched genes and the magnitude of significance, respectively.

The GO enrichment analysis showed that there were significantly enriched items in the three main categories of biological processes, cell components and molecular functions (*p* < 0.05) (Table 6). More specifically, representative biological processes include positive regulation of NF-kappa B transcription factor activity, neuromuscular synaptic transmission, positive regulation of interleukin-8 production and positive regulation of chronic inflammatory response to antigenic stimulus. Similarly, in terms of molecular functions, CNVRs are mainly enriched in nucleic acid binding, receptor activity and heat shock protein binding. The identified cellular components are significantly enriched in perinuclear region of cytoplasm, integral component of plasma membrane and cell–cell adherens junction (Figure 3a). The results of the KEGG pathway enrichment analysis are shown in Figure 3b. The figure shows the pathways with a significantly different *p*-value (*p* < 0.05). The Jinjiang horse CNVRs were highly enriched in the NF-kappa B signaling pathway, legionellosis signaling pathway and toxoplasmosis signaling pathway. Detailed information of the functional annotation is shown in Table S3.

Interestingly, our analysis results show that CNVRs in the Jinjiang horse were significantly associated with the NF-kappa B signaling pathway and the biological process of cellular heat acclimation. Based on their biofunction, two genes overlapping with CNVRs and playing important regulatory roles in the pathway were screened out, and they were selected as candidate genes for the adaptation to high temperature and humidity in Jinjiang horses. Among them, *HSPA1A* was overlapped with a Jinjiang horse breed-specific CNVR (Chr20:32247548-32491252), and *NFKBIA* was overlapped with a common CNVR (Chr1:32247548-32491252), which was found in Jinjiang horses, Zhaotong horses and Lijiang horses. In addition, two genes (*SOCS4, IL-6*) closely related to the NF-kappa B signaling pathway were added for verification by referring to previous studies [29,30].

### 3.5. Validation of Candidate Genes by qPCR

**Figure 4:**
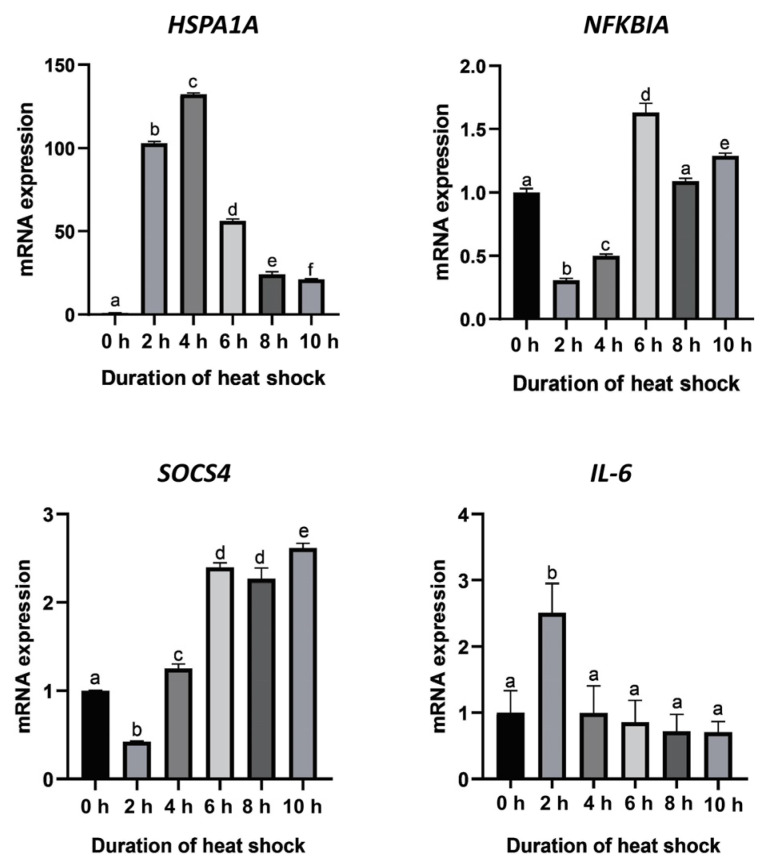
Expression levels of the four candidate genes at different heat shock durations. Means without a common superscript are significantly different (*p* < 0.05) from others.

A fluctuation in the relative expression levels of *HSPA1A* at different heat shock durations is shown in Figure 4. The result shows that the *HSPA1A* mRNA expression levels were significantly different among different heat shock durations (*p* < 0.05). Compared with the control group (heat shock for 0 h), the expression of *HSPA1A* increased rapidly after heat shock. At the fourth hour after heat shock, the expression level of *HSPA1A* significantly increased by 130-fold, reaching the highest expression level during the whole heat shock period. Subsequently, the expression level showed a continuous downward trend, and the decline tended to be gentle at 10 h after heat shock. Nevertheless, compared with the control group (heat shock for 0 h), the expression of *HSPA1A* still increased up to 20-fold.

The expression of the *NFKBIA* and *SOCS4* genes showed similar trends. The expression level of *NFKBIA* declined from the beginning of heat shock until 2 h after heat shock and then showed a gradual increase and reached the highest value at 6 h of heat shock. The expression level of *SOCS4* declined at the beginning of heat shock and reached the lowest level at 2 h after heat shock. After that, the expression level gradually increased until it reached a maximum at 10 h of heat shock, which was 2.5-fold higher than that of the control group (heat shock for 0 h). The expression of the *IL6* gene increased significantly at 2 h after heat shock and then decreased to the same level as the control group.

## 4. Discussion

The genetic variants that underlie the phenotypic diversification of horse breeds have been extensively studied in recent years. Moreover, the occurrence of CNVs in horses and their subsequent impact on phenotypic variation have not been fully investigated yet. In the present study, we described the analysis of copy number variations in Chinese indigenous horse breeds using the Illumina Equine 70 K SNP Bead Array. The distribution of CNVRs in the present study is similar to that of previous studies. The highest number of CNVRs was detected on ECA1, and ECA12 showed the largest percentage of the genome covered by CNVRs [7,18,19,21]. In addition, a particular feature possessing clusters of olfactory receptor genes was revealed with enrichment analysis, which is also observed in other mammalian genomes and was hypothesized to influence the flight response and temperament diversity in horses [24]. Similar to other studies, CNVRs less than 100 Kb accounted for the highest proportion, while CNVRs greater than 400 Kb accounted for the lowest proportion. This finding could be attributed to the fact that shorter CNVRs are more prevalent in the horse genome [7,31,32].

Compared with previous studies on horses, 131 CNVRs were newly discovered in this study, which is helpful in exploring the unique traits of Chinese indigenous horse breeds and in preserving the local genetic resources. Compared with previous studies on CNVs of Chinese indigenous horse breeds [7,21], the present study not only comprised a considerably greater number of samples but also revealed CNV fragments covering a larger proportion of the genome, in which higher CNV diversity was found and a better understanding on the distribution of CNVs in the horse populations was achieved. Regarding the average genome coverage, the differences observed among the studies may be attributed to the differences in sample sizes and genetic background, as well as the different detection platforms and methodologies applied for CNV discovery.

Among the extensive CNV studies conducted on horses, a large proportion of them focused only on CNV discovery and reported their counts and types, while few studies evaluated the potential association between CNVs and complex traits. The expression of genes can be altered by CNVs. Deletions and duplications of a part of a gene and/or a complete gene can disrupt the gene expression and potentially lead to changes in various phenotypes [33]. There have been a number of studies showing that genes overlapping with CNVs have an impact on traits of domestic animals. For example, some coat colors in horses, pigs and sheep are regulated by genes which are affected by CNVs [9,34,35]. In cattle, studies have shown that the loss and normal CNV types of the CLCN2 gene were associated with growth traits including cannon circumference, body slanting length, chest girth and body weight [36]. In our study, a common CNVR (Chr1:32247548-32491252) was detected in the three breeds of the Jinjiang horse, the Zhaotong horse and the Lijiang horse. We highlighted the *NFKBIA* gene that can regulate the inflammatory response and overlaps with this CNVR. More interestingly, some CNVRs were considered as breed-specific or identified in a few breeds, suggesting that the variability might contribute to the determination of some phenotypic characteristics and distinguishing different variety characteristics [37]. Breed-specific CNVRs were identified in the present study, and the largest number of breed-specific CNVRs was found in the Jinjiang horse. Among them, CNVR (Chr20:32247548-32491252) attracted our attention due to its overlap with the *HSPA1A* gene. The *HSPA1A* gene encodes a 70 kDa heat shock protein, which is a member of the heat shock protein 70 family [38]. Taking into account the unique environmental adaptability of Jinjiang horses, and based upon our enrichment analyses and comparison of CNV genes among different breeds and the known functions of identified genes, we highlighted certain genes that overlap with CNVRs or are associated with the heat shock response as candidate genes in this study, including *NFKBIA*, *SOCS4*, *HSPA1A* and *IL6*.

Nuclear factor-kappa B (NF-κB) is a ubiquitous transcription factor that plays an essential role in the regulation of a variety of genes involved in immune function, inflammatory response, endothelial cell activation and the control of cell growth [39,40,41]. Under normal conditions, NF-κB is rapidly activated upon response to stress responses. The NF-κB pathway is involved in the mechanisms in response to heat shock, which has been reported in a number of studies [42,43,44,45]. Induction of heat shock protein (HSP) confers protection against various forms of cellular and tissue injury, and the molecular chaperone properties of HSPs are essential to their cytoprotective effects [46]. Our results show that *HSPA1A* expression increased rapidly and greatly at the beginning of heat shock to protect cell homeostasis. Interleukin-6 (*IL6*) is a pleiotropic cytokine with significant functions in the regulation of the immune system. As a potent pro-inflammatory cytokine, *IL6* plays a pivotal role in host defense against pathogens and acute stress [47]. Previous studies have shown that the activity of NF-κB was significantly upregulated in *HSP70*-overexpressed cells and promoted the secretion of pro-inflammatory cytokines [29]. In our study, *IL6*, as a downstream pro-inflammatory cytokine of the NF-κB pathway, was significantly increased after 2 h of heat stress, indicating that NF-κB was activated and the cells produced an immune response. However, increased or deregulated expression of *IL6* may lead to excessive inflammatory responses [30]. It is well known that the NF-κB inhibitor alpha (*NFKBIA*) has great potency in suppressing NF-κB by keeping it sequestered in an inactive state in the cytoplasm [48]. Cytokines play important roles in the modulation of physiological systems. The suppressors of the cytokine signaling (SOCS) family are important immune regulators in mammals [49]. As previous studies have shown, *SOCS4*, as a suppressor of cytokine signaling, can negatively regulate the inflammatory response [29]. Our result shows that the expression level of the *NFKBIA* and *SOCS4* genes showed an increased trend at 4 h of heat shock. Previous studies have shown that the heat shock response blocked *NFKBIA* degradation and upregulated *NFKBIA* expression [50]. Coincidentally, another study showed that the stress response inhibited the dissociation of *NFKBIA* from NF-κB and the subsequent degradation of *NFKBIA*, and induction of the stress response also increased the expression of *NFKBIA* [51]. We deduce that cells prevent excessive inflammatory responses in two ways. On the one hand, the expression of *HSPA1A* can enhance the expression of the *NFKBIA* gene and inhibit the phosphorylation degradation of *NFKBIA*, and its main role is to prevent cell damage caused by excessive inflammation by inhibiting the activation of NF-κB. On the other hand, the cytokine signaling suppressor *SOCS4* is released to protect organs from injury. Specifically, *SOCS4* overexpression negatively regulated the activity of NF-κB, thereby inhibiting the inflammatory response in the present study. This may explain why the expression levels of *IL6* decreased after 2 h of heat shock.

Overall, we observed a significant increase in the expression of *IL6* during the first 2 h of heat shock, indicating that an inflammatory response was produced in the cells. Subsequently, the increased expression of *NFKBIA* and *SCOS4* suppressed inflammatory responses by inhibiting the activation of NF-κB, thereby maintaining cellular homeostasis. The specific regulatory mechanism needs to be confirmed by subsequent verification experiments, including the study on specifically expressed genes related to the high temperature adaptation of the Jinjang horse, and the association of gene expression and overlapped CNVRs. The CNVRs and the overlapped genes on ECA20 of the Jinjiang horse identified in the present study provide new clues to reveal the genetic mechanism of the adaptability to high-temperature and humidity environments in the Jinjiang horse.

## 5. Conclusions

A comprehensive genome-wide CNV map of Chinese indigenous horses was constructed using SNP genotyping data at the genomic level in the present study. There are plenty of novel and breed-specific CNVRs in Chinese indigenous horse breeds, and the results support the hypothesis that CNVs are ubiquitous in the horse genome. Candidate genes overlapping the CNVRs of the Jinjiang horse, which may be related to adaptation to high temperature and humidity, were revealed and verified with in vitro experiments. The findings of the present study provide valuable information for assessing the genetic resources of Chinese indigenous horses and shed new light on the molecular mechanism of adaptations to high-temperature and humidity environments in horses.

## Figures and Tables

**Table 1:** Sample information of ten Chinese indigenous horse breeds.

Breed Name	Number of Original Samples	Number of Samples after Quality Control	Region	Group	Climate Type
Kazakh	17	15	Xinjiang	Kazakh Horse Type	Temperate continental climate
Inner Mongolian	23	20	Inner Mongolia	Mongolian Horse Type	Temperate continental climate
Daan	26	25	Jilin Province	Mongolian Horse Type	Temperate monsoon climate
Chakouyi	34	30	Gansu Province	Hequ Horse Type	Alpine climate
Naqu	29	27	Tibet	Tibetan Horse Type	Alpine climate
Jinjiang	57	55	Fujian Province	Southwest Horse Type	Subtropical maritime monsoon climate
Zhaotong	26	25	Yunnan Province	Southwest Horse Type	Subtropical monsoon climate
Tengchong	22	22	Yunnan Province	Southwest Horse Type	Subtropical monsoon climate
Lijiang	31	28	Yunnan Province	Southwest Horse Type	Subtropical monsoon climate
Baise	36	35	Guangxi Province	Southwest Horse Type	Subtropical monsoon climate
Total	301	282	-	-	-

**Table 2:** Statistical results of CNV identification *.

Breed Name	Sample Size	Number of CNVs	Average Number of Individual CNVs	Average Length of CNVs (Kb)	Length Range of CNVs (Kb)
Baise	35	64 (24)	1.83 (0.69)	270.21	3.95~1166.66
Chakouyi	30	54 (23)	1.80 (0.77)	209.09	2.69~1166.66
Daan	25	34 (10)	1.36 (0.40)	307.60	23.50~2023.07
Inner_Mongolian	20	37 (8)	1.85 (0.40)	253.25	2.69~1269.78
Jinjiang	55	134 (72)	2.44 (1.31)	204.90	2.69~1993.59
Kazakh	15	20 (4)	1.33 (0.27)	263.03	13.92~1432.70
Lijiang	28	74 (36)	2.64 (1.29)	305.76	2.69~1886.77
Naqu	27	48 (11)	1.78 (0.41)	303.24	19.78~1607.80
Tengchong	22	45 (16)	2.05 (0.73)	275.73	19.30~1993.59
Zhaotong	25	67 (24)	2.68 (0.96)	226.83	1.06~1578.11
Total	282	577 (228)	2.05 (0.81)	261.96	1.06~2023.07

* The numbers in parentheses are breed-specific CNV counts.

**Table 3:** Statistical results of CNVR identification *.

Breed Name	Sample Size	CNVRs	Gain	Loss	Mixed	Average Number of Individual CNVRs
Baise	35	52 (21)	36 (11)	14 (9)	2 (1)	1.49 (0.60)
Chakouyi	30	50 (24)	32 (10)	17 (13)	1 (1)	1.67 (0.80)
Daan	25	29 (9)	19 (3)	10 (6)	0 (0)	1.16 (0.36)
Inner_Mongolian	20	33 (8)	22 (1)	11 (7)	0 (0)	1.65 (0.40)
Jinjiang	55	113 (64)	79 (38)	33 (25)	1 (1)	2.05 (1.15)
Kazakh	15	19 (6)	15 (5)	4 (1)	0 (0)	1.27 (0.40)
Lijiang	28	63 (31)	38 (12)	23 (18)	2 (1)	2.25 (1.11)
Naqu	27	42 (11)	26 (3)	15 (8)	1 (0)	1.56 (0.41)
Tengchong	22	38 (15)	30 (9)	7 (5)	1 (1)	1.73 (0.68)
Zhaotong	25	56 (22)	38 (13)	17 (9)	1 (0)	2.24 (0.88)
Total	282	495 (211)	335 (105)	151 (101)	9 (5)	1.76 (0.75)

* The numbers in parentheses are breed-specific CNVR counts.

**Table 4:** Descriptive statistics of CNVRs on equine autosomes.

Chr	Length of Chromosomes (Mb)	Number of CNVRs	Length of CNVRs (bp)	Percentage (%)	Average Length of CNVRs (bp)
1	188.26	34	6,227,042	3.31%	183,148.29
2	121.35	16	1,984,086	1.64%	124,005.38
3	121.35	17	2,359,505	1.94%	138,794.41
4	109.46	24	2,907,700	2.66%	121,154.17
5	96.76	7	458,305	0.47%	65,472.14
6	87.23	8	556,343	0.64%	69,542.88
7	100.79	9	1,237,197	1.23%	137,466.33
8	97.56	9	1,463,869	1.50%	162,652.11
9	85.79	4	753,364	0.88%	188,341.00
10	85.16	8	1,117,790	1.31%	139,723.75
11	61.68	3	348,307	0.56%	116,102.33
12	36.99	11	5,799,518	15.68%	527,228.91
13	43.78	2	434,027	0.99%	217,013.5
14	94.6	4	653,789	0.69%	163,447.25
15	92.85	7	979,123	1.05%	139,874.71
16	88.96	2	701,010	0.79%	350,505.00
17	80.72	7	832,567	1.03%	118,938.14
18	82.64	14	2,861,413	3.46%	204,386.64
19	62.68	1	1,128,766	1.80%	1,128,766.00
20	65.34	6	698,764	1.07%	116,460.67
21	58.98	4	658,611	1.12%	164,652.75
22	50.93	2	973,451	1.91%	486,725.5
23	55.56	3	504,860	0.91%	168,286.67
24	48.29	3	520,059	1.08%	173,353.00
25	40.28	4	1,067,167	2.65%	266,791.75
26	43.15	13	2,429,584	5.63%	186,891.08
27	40.25	4	602,367	1.50%	150,591.75
28	47.35	2	310,645	0.66%	155,322.5
29	34.78	4	222,270	0.64%	55,567.5
30	31.4	2	198,567	0.63%	99,283.5
31	26	5	750,488	2.89%	150,097.6
Total	2280.92	239	41,740,554	1.83%	174,646.67

**Table 5:** Comparison of CNVRs identified in this study with those identified in ten previous studies.

Study	Platform	Breed	Sample	CNVR Count	CNVR Range (kb–Mb)	Genome Enrichment %	Reference Genome	Overlapped CNVR Count with the Present Study
Doan et al. (2012)	Array CGH	15	16	775	0.2–3.5	3.7	EquCab 2.0	22
Metzger et al. (2013)	Illumina Equine 70 K SNP BeadChip	17	717	50	0.5–0.9	1.7–22.0	EquCab 2.0	28
Dupuis et al. (2013)	Illumina Equine 70 K SNP BeadChip	4	447	478	0.1–2.7	2.3	EquCab 2.0	24
Ghosh et al. (2014)	Array CGH	16	38	258	1–2.5	1.15	EquCab 2.0	19
Wang et al. (2014)	Array CGH	6	6	353	6.1–0.5	0.61	EquCab 2.0	11
Kader et al. (2016)	Illumina Equine 70 K SNP BeadChip	3	96	122	0.2–2.2	0.8	EquCab 2.0	14
Ghosh et al. (2016)	Array CGH	NA	63	245	NA	NA	EquCab 2.0	20
Schurink et al. (2018)	Axiom Equine Genotyping Array (670,796 SNPs)	1	222	5350	0.12–1.03	11.2	EquCab 2.0	22
Solé et al. (2019)	Axiom Equine Genotyping Array (670,796 SNPs)	8	1755	939	1–21.3	NA	EquCab 2.0	80
Corbi-Botto et al. (2019)	Illumina GGP Equine 70 K	1	24	87	0.5–2	0.6	EquCab 2.0	10
Present study	Illumina Equine 70 K SNP BeadChip	10	300	239	1.06–2.44	1.83	EquCab 3.0	-

**Table 6:** Functional enrichment analysis of CNVR-overlapping genes in the Jinjiang horse.

Category	ID	Term	Counts	*p*-Value	Genes
KEGG	ecb05134	Legionellosis	4	3.33 × 10^−3^	*HSPA1A, TNF, NFKBIA*
KEGG	ecb03040	Spliceosome	5	5.81 × 10^−3^	*HSPA1A, HNRNPC, LSM2*
KEGG	ecb04064	NF-kappa B signaling pathway	4	1.02 × 10^−2^	*LTB, TNF, NFKBIA*
KEGG	ecb05145	Toxoplasmosis	4	1.45 × 10^−2^	*HSPA1A, TNF, NFKBIA*
KEGG	ecb05166	Human T-cell leukemia virus 1 infection	5	1.86 × 10^−2^	*APC2, LTA, NFKBIA*
GO_BP	GO:0051092	Positive regulation of NF-kappa B transcription factor activity	6	1.82 × 10^−3^	*NFKBIA, TRAPPC9, TNF, HSPA1A*
GO_BP	GO:0032757	Positive regulation of interleukin-8 production	3	1.25 × 10^−2^	*TNF, HSPA1A*
GO_BP	GO:0007274	Neuromuscular synaptic transmission	3	1.25 × 10^−2^	*FCHSD2, CHRNB3, CHRNA6*
GO_BP	GO:0002876	Positive regulation of chronic inflammatory response to antigenic stimulus	2	1.31 × 10^−2^	*LTA, TNF*
GO_MF	GO:0003676	Nucleic acid binding	14	1.35 × 10^−2^	*DAZAP1, CIRBP, RNASE6, ZNF219*
GO_MF	GO:0004872	Receptor activity	6	1.48 × 10^−2^	*NCR3, LRP1, SLC20A2, CADM2*
GO_MF	GO:0005102	Receptor binding	7	3.02 × 10^−2^	*NCR3, CADM2*, *HSPA1A*
GO_MF	GO:0031072	Heat shock protein binding	3	3.16 × 10^−2^	*NFKBIA, HSPA1A*
GO_CC	GO:0048471	Perinuclear region of cytoplasm	11	9.40 × 10^−3^	*APC2, PDE2A, M6PR, HSPA1A*
GO_CC	GO:0005887	Integral component of plasma membrane	18	1.42 × 10^−2^	*LRP1, KCNK9, CADM2*
GO_CC	GO:0005913	Cell–cell adherens junction	7	2.24 × 10^−2^	*NCR3, BAIAP2L1*, *HSPA1A*

## Supplementary Materials

The following supporting information can be downloaded at: https://www.mdpi.com/article/10.3390/genes13040603/s1, Table S1: The primer sequences for qPCR; Table S2: CNVs and CNVRs identified in this study; Table S3: Gene annotation and functional enrichment analysis of 113 CNVRs in Jinjiang horses.
